# Virulence Genes Content and Antimicrobial Resistance in *Escherichia coli* from Broiler Chickens

**DOI:** 10.1155/2014/195189

**Published:** 2014-11-24

**Authors:** Moemen A. Mohamed, Mostafa A. Shehata, Elshimaa Rafeek

**Affiliations:** ^1^Poultry Diseases Department, Faculty of Veterinary Medicine, Assiut University, Assiut 71515, Egypt; ^2^Regional Veterinary Laboratory, Veterinary Research Institute, Assiut, Egypt

## Abstract

A total of 121* E. coli* strains were isolated from broiler chickens (96 extraintestinal pathogenic (ExPEC) strains from diseased broiler chickens and 25 avian fecal* E. coli* (AFEC) from healthy ones). Ten of the isolates (6 from diseased chickens and 4 from healthy birds) were serogrouped and 25 were examined for 4 virulence markers (*tsh*,* papC*,* colV*, and* iss* genes) as well as for their antimicrobial resistance. Five strains were nontypable and the rest were serotyped as follows: O86:K61 (2/5), O78:K80 (1/5), and O128:K67 (1/5) were recovered from diseased chickens, while O111:K58 strain (1/4) was isolated from healthy ones. The* iss *gene was found in 72.2% of the examined ExPEC strains in contrast to zero percentages (0%) in the AFEC strains, which may serve as a good marker for distinguishing APEC and its knocking out may help in creation of candidate vaccine that may prove sucess in elimination of infections in broiler chickens. Antimicrobial resistance patterns revealed a complete resistance to gentamicin, pefloxacin, amoxicillin, and enrofloxacin among examined strains followed by varying degrees of resistance for the rest of tested agents. The highest resistance was recorded against norfloxacin, in 24 isolates (96%), in contrast to the lowest resistance was recorded against colistin sulphate, in 14 strains (56%). These findings suggest the need for the prudent use of antimicrobials with broiler chickens and act as a warrant for the possibility of avian sources to transmit these resistant isolates to humans.

## 1. Introduction

Avian pathogenic* Escherichia coli* (APEC), a subgroup of extraintestinal pathogenic* E. coli* (ExPEC), enters through different routes including respiratory and genital tracts and causes various extraintestinal diseases collectively termed as colibacillosis in chickens, which are responsible for significant economic losses in the chicken industry [1–3]. The pathogenicity of avian pathogenic* E. coli* (APEC) that permits certain intestinal commensal* E. coli* to become APEC and infect extraintestinal niche is the result of the expression of several putative virulence factors [4–7].

These virulence traits can be categorized as adhesion, iron acquisition, hemolysis, protection from bactericidal host factors, and toxin production [4, 8]. Temperature-sensitive haemagglutinin (*tsh*) plays a role in the colonization of air sacs [9]; P-fimbriae (pap) are important in the later stages of infection for the adhesion to internal organs [10]. The bacterial resistance to the complement (*colV*) giving resistance to phagocytosis [11] has been associated with APEC strains [12] and the increased serum survival (*iss*) gene [13] is known to be associated with serum resistance and is considered the most significantly associated gene with APEC strains [14].

Antimicrobial therapy is one of the primary control measures for reducing the morbidity and mortality caused by APEC infections. Since the indiscriminate use of antimicrobials leads to the selection of resistant isolates, they need to be used prudently in order to preserve their therapeutic usefulness in both animals and humans [15].

The precise set of virulence factors possessed by ExPEC and avian fecal* E. coli* (AFEC) isolated from healthy birds is not clearly established in isolates of Assiut Province; without specific information about ExPEC, it is difficult to predict the success of colibacillosis control schemes. So, the purpose of this study was to characterize both groups occurring in a chicken broiler in commercial farms in diseased and healthy ones using microbiological and molecular techniques.

## 2. Materials and Methods

### 2.1. Sample Collection

Two hundred and seventy-six samples from freshly dead birds (liver, heart blood, lung, trachea, and sinus from cases of swollen head syndrome) and 27 samples from apparently healthy broiler chickens (cloacal swabs) were collected from 11 different poultry farms located in different geographic areas in Assiut Governorate, Egypt, in year 2011. Samples were immediately transported in an ice tank to the laboratory, being stored at 3°C ± 1°C for no longer than 1 h before analysis.

### 2.2. Bacterial Isolation and Identification

The respective specimens from extraintestinal places were firstly incubated on nutrient broth at 37°C for 24 h and then subcultured on the eosin methylene broth for the same period and temperature. A loopful of the broth was subcultured onto MaCconkey agar (Oxoid) and incubated aerobically for 24 h at 37°C. From each plate, a single colony of typical morphology was picked and subcultured onto eosin methylene blue (EMB) for purity and biochemical testing. Suspect* E. coli* colonies displaying typical morphology were identified biochemically according to the protocol of Betty et al. [18].* E. coli* isolates were stored at −80°C in brain heart infusion (BHI) broth containing 25% glycerol until used.

### 2.3. Determining the Serogroups

Serogroups were identified using the slide agglutination test (Edwards and Ewing [19]). The complete commercial kit for* E. coli* serotyping was done at the Department of Food Hygiene Control, University of Benha, Qalyubia, Egypt.

### 2.4. Virulence Genotyping

Twenty-five strains (18 ExPEC, 7 AFEC) were examined for the presence of the 4 virulence genes (*tsh*,* colV*,* papC,* and* iss*) using PCR. Crude DNA extracts were obtained by using Wizard Genomic DNA Purification kit. The resulting DNA template was stored at −20°C until further use.

A single PCR assay was used to detect each one of the 4 virulence genes [16, 17]. Primers used for PCR are listed in Table 1. The PCR conditions were as follows: 94°C for 3 min, followed by 30 cycles of 94°C for 1 min, annealing for 1 min at 72°C for at least 30 s according to the size of the amplified fragment (1 min/kb), and then a final extension at 72°C for 10 min. All samples were subjected to horizontal gel electrophoresis in 2% agarose, and amplicon sizes were determined by comparison to the Hi-Lo DNA marker obtained from Minnesota Molecular Inc. (MN). An isolate was considered to contain a gene of interest if it produced an amplicon of the expected size (Table 1).

### 2.5. Antimicrobial Susceptibility Testing

Twenty-five strains (18 ExPEC, 7 AFEC) were examined for the antimicrobial sensitivity using a Kirby-Bauer disk diffusion assay according to the standards and interpretive criteria described by CLSI [20]. The following antibiotics were used: cefotaxime (CTX), 30 ug; gentamicin (GEN), 10 ug; streptomycin (STR), 10 ug; sulfamethoxazole-trimethoprim (SXT), 23.75/1.25 ug, and oxytetracycline (OXT), 30 ug; colistin (CT), 25 ug; doxycycline (DO), 30 ug; norfloxacin (Nor), 10 ug; pefloxacin (PF), 5 ug; amoxicillin (AM), 25 ug; neomycin (N), 30 ug; flumequine (FL), 30 ug; and enrofloxacin (EN), 10 ug.

## 3. Results

### 3.1. Prevalence of* E. coli* within the Examined Birds

Ninety-six strains (34.8%) were isolated from extraintestinal origins in diseased cases in contrast to 25 strains (92.6%) which were isolated from cloacal swabs of healthy birds.

### 3.2. Serological Characterization

Ten of the isolated strains were serotyped. Five were nontypeable and the rest belonged to 4 serotypes: O86:K61 (2/5), O78:K80 (1/5), and O128:K67 (1/5) were recovered from extraintestinal origins in diseased birds, while O111:K58 strain (1/5) was isolated from intestinal contents of healthy ones.

### 3.3. Detection of Virulence Genes

**Figure 1:**
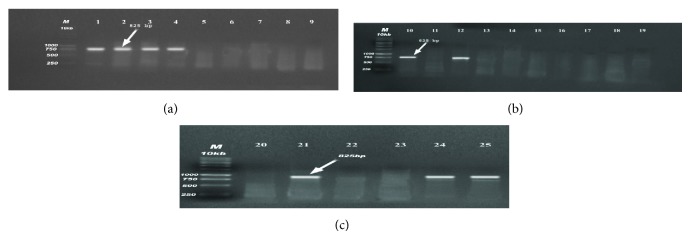
(a, b, c) Detection of* tsh* gene in samples. Positive samples produce band (825 bp); lane M: 1 Kb DNA Ladder; lanes 1, 2, 3, 4, 10, 12. 21, 24, and 25 were positive samples.

**Figure 2:**
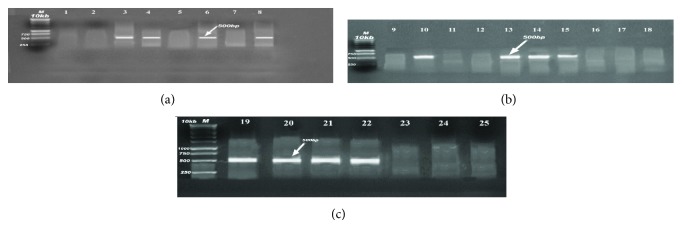
(a, b, c) Detection of* papC* gene in samples; positive samples produce band (500 bp); lane M: 1 Kb DNA Ladder; lanes 3, 4, 6, 8, 10, 13, 14, 15, 19, 20, 21, and 22 were the positive samples produced band (500 bp).

**Figure 3:**
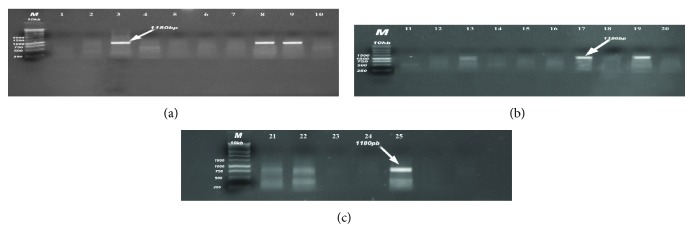
(a, b, c) Detection of* colV* plasmid gene in samples; positive samples produce band 1180 bp; lane M: 1 Kb DNA Ladder; lanes 3, 8, 9, 17, 19, and 24 were positive samples.

**Figure 4:**
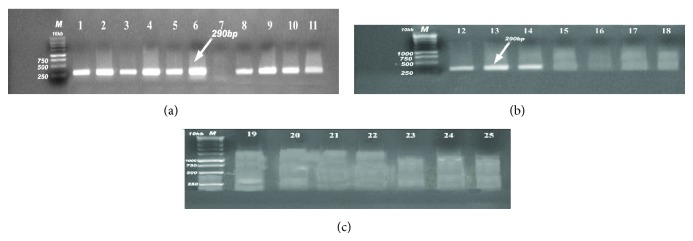
(a, b, c) Detection of* iss* gene in samples; positive samples produce band 290 bp; lane M: 1 Kb DNA Ladder; lanes 1–6 and 8–14 were positive samples.

PCR analysis of the 25* E. coli* isolates was performed to detect virulence genes to assign them to specific pathotypes (Figures 1, 2, 3, and 4). The 4 virulence genes were found among the 18 examined strains of diseased birds with different percentages: 72.2% harboring* iss*, 44.4% positive to* papC*, 33.3% to* tsh*, and 27.8% to* colV*, in contrast to 57.1% which were positive* papC*, 42.9% to* tsh*, 14.29% to* colV,* and zero percentages to* iss* gene (0%) in strains recovered from apparently healthy broilers chickens (Table 2).

### 3.4. Antimicrobial Resistance

**Figure 5:**
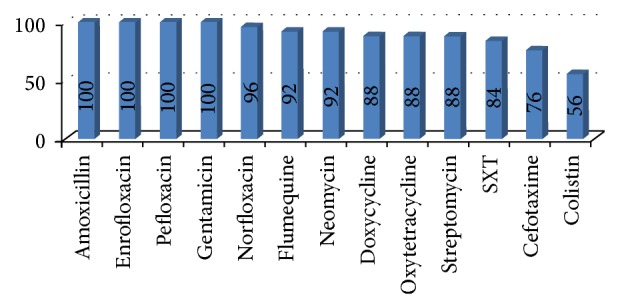
Antimicrobial resistance percentages of* E. coli* isolates to 13 antimicrobials tested individually using disc diffusion tests.

A total of 25* E. coli* strains were tested for their resistance to thirteen antimicrobial drugs using the conventional agar disc diffusion method. The results showed a high degree of resistance to the examined antimicrobial agents; 25 (100%) isolates showed complete resistance to gentamicin, pefloxacin, amoxicillin, and enrofloxacin, 24 (96%) isolates were resistant to norfloxacin, 23 (92%) isolates were resistant to neomycin and flumequine, 22 (88%) isolates were resistant to oxytetracycline and streptomycin and doxycycline, 21 (84%) isolates were resistant to sulfamethoxazole and trimethoprim (SXT), 19 (76%) isolates were resistant to cefotaxime, and 14 (56%) isolates were resistant to colistin sulphate (Figure 5).

## 4. Discussion

Four serogroups were detected (O78, O86, O128, and O111). The most common isolated serogroups from the diseased cases were O78 and O86 which represented 60% of the obtained serogroups followed by O128 (20%). In contrast to the obtained seorgroup isolated from healthy cases was O111 (20%), that recorded often with human severe gastroenteritis [21]. The fact that half of the examined strains in this study were nontypeable confirms the need to use other characterization methods to describe the pathotype.

In the current study, we used a PCR for screening a collection of 25* E. coli* isolates recovered from diseased and clinically healthy broilers in Assiut, Egypt. The results revealed that the frequency of the virulence genes possession was different from those previously reported [16, 17, 22]. The* tsh* gene was detected in 85.3% of the APEC analyzed by Janßen et al. [17], in 39.5% of the strains analyzed by Delicato et al. [22], and in 53.3% of the strains analyzed by Ewers et al. [16]. In this study, the* tsh*+ strains frequency remained at 28%. Similarly, Ewers et al. [16] detected 82.7% of strains positive to the* iss* gene, Delicato et al. [22] reported 38.5%, and this study detected only 33.3% in extraintestinal isolates and 42.9% in intestinal strains.

The* papC* gene is localized to pathogenicity islands (PAIs) that are chromosomal in location [17]. Rodriguez-Siek et al. [23] reported that the* papC* gene was more likely to be found among APEC (44.1%) than among AFEC (9.5%). There is a provocative difference between the isolates studied here and their counterparts; that is, about 57.1% of the AFEC harbored* papC* gene in contrast to 44,4 for ExPEC. Its high prevalence in* E. coli* isolated from apparently healthy birds is perhaps due to the fact that these birds were infected but preclinically affected or their environments contained a heavy load of APEC which passed through the birds without causing infection. Nevertheless, future studies will be required to explain this anomaly adequately.

*iss* gene has been detected at a higher percentage in extraintestinal strains of the diseased birds that reached 72.2% in contrast to zero percentages in the intestinal or fecal ones that may reflect its importance in their pathogenicity. In the United States, 85.4% of APEC strains isolated from lesions of birds clinically diagnosed with colibacillosis were positive for* iss* (80.5%) [14]. Moreover,* iss *was found in 82.7% of APEC strains isolated from chickens with colisepticemia in Germany [16]. However, Delicato et al. [22] obtained a lower prevalence for* iss* from APEC isolated from poultry with colibacillosis in Brazil, with an incidence of 38.5%. However, the frequency for* iss* gene was 18.7% of the fecal isolates from healthy birds [23], whereas in this study it was not detected in isolates recovered from healthy birds.

Antimicrobial agents have been widely used in poultry to treat infections caused by a variety of bacterial pathogens. However, this widespread use of large quantities of antimicrobials in poultry in some countries, including Egypt, often without professional consultation or supervision, is problematic [24].

In this study, we showed a high rate of resistance to the majority of the examined antimicrobial agents. 100% of the tested* E. coli* isolates showed resistance against gentamicin, pefloxacin, amoxicillin, and enrofloxacin followed by considerable resistance to the rest of the examined agents. In comparison, the obtained data of antimicrobial resistance in this study, with several published reports indicated that most recovered* E. coli* poultry are antimicrobial-resistant with equal or lower percentages as recorded in China [25], United States [26], Korea [27], United Kingdom [28], and Australia [29] as well as from* E. coli* strains isolated from intensively farmed and free-range poultry [30].

The most striking finding from this study was widespread resistance to gentamicin and fluoroquinolones (enrofloxacin, pefloxacin, and norfloxacin). This high presence of Quinolone-Resistant* Escherichia coli* (QREC) from the broiler chickens probably is due to overuse of enrofloxacin in therapeutic and prevention purposes in Assiut province. Fluoroquinolones are critically important for treating serious infections by* E. coli* in humans, and continued surveillance is required to detect emerging fluoroquinolone-resistant phenotypes [31]. Cephalosporins are the first-line antimicrobials for treating human bacterial infections [32]. The high percentage of strains resistant to this drug in the present result is a critical result and indicates that* E. coli* isolates of poultry origin could be the cause of treatment failure in either poultry or human.

To sum up, our findings clearly show that* E. coli* is strongly prevalent in conventional poultry production systems. ExPEC isolates varied from AFEC in their virulence gene content; that is, ExPEC possessed* iss* gene with a percentage of 72.1% and* colV* gene with a percentage of 27.7% in contrast to zero percentages for* iss* gene and 14.3% for the* colV* gene in AFEC isolates. A better clarification of the importance of virulence genes in* E. coli* isolates may be gained by investigation on the in vivo and in vitro expression of virulence genes.

Secondly, the high levels of antimicrobial resistance in* E. coli* strains, including resistance to clinically valuable antimicrobials, suggest that* E. coli* from poultry in Assiut Province, Egypt, may play a considerable role as a reservoir for resistance genes and be a key source for the transfer of resistance to other major human pathogens that emphasize the need for more rigorous surveillance and improved farming practices (including the strict regulation of antibiotic usage), which can reduce the carriage of antibiotic-resistant bacteria on foods and thereby minimize the likelihood of horizontal gene transfer of mobile antibiotic resistance genes to other bacteria through the farm and processing plants.

## Figures and Tables

**Table 1:** Showing primers that were used in PCR reaction.

Detected gene	Primer sequence (5′-3′)	PCR product size (bp)	Reference
TSH-F-15	ACT ATT CTC TGC AGG AAG TC	825	[16]
TSH-R-15	CTT CCG ATG TTC TGA ACG T		

PapC-F-15	TGA TAT CAC GCA GTC AGT AGC	500	[16]
PapC-R-15	CCG GCC ATA TTC ACA TAA		

Colv-F-15	TGG TAG AAT TGT GCC AGA GCA AG	1180	[17]
Colv-R-15	GAG CTG TTT GTA GCG AAG CC		

iss-F-15	ATG CAG GAT AAT AAG ATG AAA	290	[16]
iss-R-15	CTA TTG TGA GCA ATA TAC A		

**Table 2:** Distribution of virulence-associated genes in strains recovered from diseased and healthy birds.

Virulence gene	Strains recovered from diseased birds (*n* = 18)		Strains recovered from healthy birds (*n* = 7)	
	*N*	%	*N*	%
*tsh *	6	33.3	3	42.9
*colV *	5	27.8	1	14.29
*papC *	8	44.4	4	57.1
*iss *	13	72.2	0	0
